# Environmental Factors Influence Language Development in Children with Autism Spectrum Disorders

**DOI:** 10.1371/journal.pone.0004683

**Published:** 2009-04-09

**Authors:** Marine Grandgeorge, Martine Hausberger, Sylvie Tordjman, Michel Deleau, Alain Lazartigues, Eric Lemonnier

**Affiliations:** 1 Université de Rennes 1, Ethos, UMR 6552 CNRS – Ethologie animale et humaine, Rennes, France; 2 Department of Child and Adolescent Psychiatry, CHU Guillaume Régnier, Rennes, France; 3 Université Rennes 2, CRPCC, EA 1285, Centre de recherches en psychologie, cognition et communication, Rennes, France; 4 Service de Pédopsychiatrie, CHU de Brest, Hôpital de Bohars, Bohars, France; James Cook University, Australia

## Abstract

**Background:**

While it is clearly admitted that normal behavioural development is determined by the interplay of genetic and environmental influences, this is much less the case for psychiatric disorders for which more emphasis has been given in the past decades on biological determinism. Thus, previous studies have shown that Autistic Spectrum Disorders (ASD) were not affected by parental style. However, animal research suggests that different behavioural traits can be differentially affected by genetic/environmental factors.

**Methodology/ Principal Findings:**

In the present study we hypothesized that amongst the ASD, language disorders may be more sensitive to social factors as language is a social act that develops under social influences. Using the Autism Diagnostic Interview-Revised, we compared the early characteristics of sensori-motor and language development in a large sample of children with ASD (n = 162) with parents belonging to different levels of education. The results showed that children raised by parents with a high level of education displayed earlier language development. Moreover, they showed earlier first words and phrases if their mother was at a high level of education, which reveals an additional gender effect.

**Conclusions/Significance:**

To our knowledge this study may trigger important new lines of thought and research, help equilibrate social and purely biological perspectives regarding ASD and bring new hopes for environmentally based therapies.

## Introduction

Although the nature/nurture debate may seem to belong to past history, the question of how genetic/experiential factors affect behavioural development remains very vivid [1]. Both genetic and environmental factors are involved in the determinism of aspects like temperament, but their relative weights may vary according to the trait being considered [e.g. 2]. As mentioned by Gosling [3], animal studies are very useful as they can reveal the interplay between different factors. Thus, horses with highly sensitive phenotypes [e.g. 4] may develop abnormal behaviour (such as stereotypies) as a consequence of unfavourable environmental conditions [e.g. 5] (See [6], [7] for reviews).

These animal studies provide useful framework to study normal and pathological behaviours of humans as a result of such interplay. Thus, twin studies show that parenting influences children's prosocial behaviours and acts as a “modulation” of genetic influences. This is especially true in the case of psychiatric disorders: despite a strong genetic basis [8], schizophrenia can been shown to be influenced by parenting profiles [9] as well as by factors such as an infectious disease during mid-pregnancy [10]. The weights attributed to genetic/environmental factors by authors are also often subject to variations along with “science history”, especially where psychiatric disorders are concerned [11].

Thus, Autistic Spectrum Disorders (ASD) characterized by social and communication deficits and repetitive or stereotypic behaviour [12] have been for a long while attributed to environmental factors such as mothering (i.e. “refrigerator mother” [13]) or diseases (e.g. congenital rubella [14]). After reacting against the theory of lack of maternal affection during the '50s and '60s, research radically turned towards a neural and cognitive hypothesis [e.g. 15]. Since developments of genetic and neurology technologies during the '90s, more emphasis has been clearly given to biological (i.e. genetic) bases for these disorders (see [16] for review). The well-known social withdrawal of children with ASD has been attributed lately to deficits in the *superior temporal sulcus* voice selective regions: hearing and processing impairments based on developmental biological deficits could lead to social withdrawal [17].

Here again, animal studies suggest a much more complex situation. Thus, social experience is crucial for the development of the central auditory area in young songbirds [18], [19]. More interestingly, social segregation may induce the same deficits in a central auditory area as physical isolation and/or auditory deprivation [20]. Direct social contact with adults and the quality of interactions may strongly influence both vocal and perceptual development both in birds and humans [21], [22].

Researchers generally acknowledge that ASD are not affected by parental style but one can wonder whether as in animals [2], different behavioural traits are differently affected by genetic/environmental factors. The above mentioned results suggest that language development may be strongly affected by social factors and language abnormalities are the first observed deficit observed in more than half the families of children with ASD [23], [24].

Normally language development of children raised by parents with a high level of education is faster than that of children raised by parents with a low level of education (e.g. lexical richness [25]). In addition, parents' monitoring of language interactions with children differs according to their socioeconomic status [e.g. 26]. Moreover, mothers and fathers appear to influence children in different ways [27].

In the present study, we hypothesized that parental characteristics influenced language development in children with ASD. We compared early characteristics of language development (using *Autism Diagnostic Interview-Revised*, ADI-R; [28]) for a large sample of children with ASD of parents with different levels of education. These data were compared on similarly acquired items on other non language variables. Our results demonstrate for the first time that parental characteristics (i.e. level of education and gender) can influence language development of children with ASD. This finding may trigger important new lines of thought and research (on the mechanisms underlying this influence, stimulate investigations on exact links between parents' level of education and their language inputs to their children), help equilibrate social and purely biological perspectives regarding ASD and bring new hopes for environmentally based therapies.

## Methods

### Children

All children were recruited from the “Centre de Ressource Autisme”, Brest, France (n = 162, 135 males and 27 females, mean age at assessment, in months ±SD (min–max): 98±54 (37–373); other demographic data in Table 1). They all met the criteria of the *Diagnostic and Statistical Manual of Mental Disorders* 4^th^ edition [12] and *International Classification of Diseases* [29] for ASD. All the recruited children were French natives, lived in intact families, were physically healthy and were at least 33 months old.

**Table 1:** Characteristics of the participants and both their mothers and their fathers.

N	162
**Gender**	
Male	135 (83.3%)
Female	27 (16.7%)
**Variables** (Mean±SD ; min–max)	
Age at assessment (months)	98±54 (37–373)
Age at birth (weeks)	38.7±2.8 (28–42)
Height at birth (cm)	49.5±3.1 (36–57)
Weight at birth (g)	3290±675 (1220–4770)
**Level of education of parents**	
Low level of education	
Mother	53 (32.7%)
Father	64 (39.5%)
Mid level of education	
Mother	26 (16.1%)
Father	26 (16.1%)
High level of education	
Mother	83 (51.2%)
Father	72 (44.4%)

### Parents

The level of education of each parent was scored independently (Table 1). According to the French INSEE 2003 classification, three categories were considered: (1) low level of education (*low education status* or *LES mother* and *LES father*; a professional schooling or no education), (2) mid level of education (*mid education status* or *MES mother* and *MES father*; high school and first years at college) and (3) high level of education (*high education status* or *HES mother* and *HES father*; completed college and graduate school). Mothers and fathers could have different or similar levels of education.

### Measures

Behavioural assessments were performed using the Autism Diagnostic Interview–Revised (ADI-R) for the children with ASD [28]. The ADI-R, an extensive, semi-structured parental interview, was conducted by trained psychiatrists and administered to the parents together. As both parents responded together, their answers were not independent and the child's score correspond to their common joined response. The ADI-R scale assessed the three major domains of autistic impairments: reciprocal social interactions, verbal and non-verbal communication, stereotyped behaviours and restricted interests. Based on direct clinical observation of each child by independent child psychiatrists, a diagnosis of ASD was made according to the DSM-IV [12] and ICD-10 [28] criteria and was confirmed by the ADI-R ratings.

Parents were asked questions about their children's language and sensori-motor development.

#### Language criteria used were

(a) Age of *first single words* (in months, *first single words* refer to words used repeatedly and consistently for the purpose of communication with reference to a particular concept, object or event and keep out “dad” and “mum”; children were considered as *delayed* when they used their *first single words* after 24 months old and as normal or *non delayed* when they used their *first single words* before 24 months old). (b) Age of *first phrases* (in months, *first phrases* must be consist of two words, one of which must be a verb and keep out attribute-noun combinations nor echolalic speech nor phrases that might have been learned as a single word to convey a single meaning; children were considered as *delayed* when they used their *first phrases* after 33 months old and as normal or *non delayed* when they used their *first phrases* before 33 months old). (c) *Overall level of language* used by the children was coded in two categories: they either possessed sufficient verbal skills (daily, functional use of three-word phrases that sometimes included a verb) or they did not (no functional use, mostly single words phrases or fewer than five words used on a daily basis). Finally (d) *abnormality of development evident at or before 36 months* ; each child was given a score that added (1) the age when parents first noticed something was not quite right in their child's language, relationships or behavior (if observed <36 months, score 1), (2) the age when abnormalities first became evident (if observed <36 months, score 1), (3) the interviewer's judgement on the age when developmental abnormalities probably first became manifest (if observed <36 months, score 1), (4) the age of the first single words uttered (if observed >24 months, score 1), and (5) the age of the first phrases uttered (if observed >33 months, score 1). The higher is the score, the higher is the abnormality *of development evident at or before 36 months*.

#### Sensori-motor criteria used were

(a) *Age of sitting unaided on flat surface* (in months; the age when the child first sat, without support, on a flat surface. Children were considered as *delayed* when they first sat after 8 months old and as normal or *non delayed* when they first sat 8 months old). (b) *Age of walking unaided* (in months; the age when the child walked without holding on. Children were considered as *delayed* when they walked unaided after 18 months old and as normal or *non delayed* when they walked unaided before 18 months old). (c) *Age of bladder control acquisition during daytime* (in months; the age when the child was first dry for 12 months without accidents), (d) *Age of bladder control acquisition during the night* (in months; the age when the child was first dry for 12 months without accidents). Finally, (e) *age of bowel control acquisition* (in months; the age when the child was first continent for 12 months without accidents).

All the data are confirmed by the health card of each child, a medical document filled out at each stage of the life (e.g. weight, height, age of the first walk, diseases). Verbal informed consent was given by parents and the protocol was approved by the ethics committee of Bicêtre Hospital.

### Statistical analyses

The analyses were conducted in four steps, using Minitab© software and an accepted p level of 0.05. Kruskal-Wallis tests compared ages of *sitting unaided on flat surface*, *walking unaided*, *bladder control acquisition during daytime*, *bladder control acquisition during the night*, *age of bowel control acquisition*, *first single words, first phrases* according to the three levels of education of both mothers and fathers. Post hoc pair-wise comparisons were then applied using Mann–Whitney U-tests. Chi-square tests assessed the relationships between the three levels of education of both mothers and fathers and the following qualitative variables: *first single words* and *first phrases* (*non-delayed* and *delayed* children). ANOVA test and post hoc Tukey's test assessed the relationships between the three levels of education of both mothers and fathers and the quantitative date of *abnormality of development evident at or before 36 months* (scale with six levels coded 0 to 5). Binary logistic regression assessed the relationships between the *overall level of language* and the three levels of education of both mothers and fathers, taking into account the age of children at assessment. Factors were used both in independence and in interaction (age×level of education).

## Results

A clear influence of the educational levels of parents appeared on language development while no such effect was observed on sensori-motor development.

### Language development

#### Age of first single words

One hundred and forty-eight children (91.4%) had used their *first single words* and this had occurred on average at 26.4±15.5 months (min: 6; max: 84). Seventy-five children (46.3%) uttered their *first single words* before 24 months (i.e. *non delayed*) and 73 children (41.1%) uttered their *first single words* after 24 months (i.e. *delayed*). Fourteen children (8.6%) of the cohort had not yet pronounced their *first single words* when they were assessed even though they were 82.0±68.3 months old (min: 37; max: 309) (Table 2).

**Table 2:** Range, mean age ±SD of *first single words* and *first phrases* pronounced by children, the number and percentage of associated categories (*delayed*, *non delayed* and *not achieved*) according to the level of education of mothers and fathers.

	Mothers' level of education			Fathers' level of education			TOTAL
	Low level of education	Mid level of education	High level of education	Low level of education	Mid level of education	High level of education	
**Age of ** ***first single words*** ** (months)**							
Mean±SD	32.2±18.5	23.0±9.8	24.0±14.0	26.4±15.5	28.9±16.3	24.1±10.8	**26.4±15.5**
Min–Max	8–84	12–48	6–72	6–84	8–84	9–42	**6–84**
*Non delayed* (before 24 months old)	18 (11.1%)	14 (8.6%)	43 (26.6%)	75 (46.3%)	23 (14.2%)	11 (6.8%)	**75 (46.3%)**
*Delayed* (after 24 months old)	30 (18.5%)	11 (6.8%)	32 (19.8%)	73 (45.1%)	34 (21.0%)	12 (7.4%)	**73 (45.1%)**
*Not achieved*	5 (3.1%)	1 (0.6%)	8 (4.9%)	14 (8.6%)	7 (4.3%)	3 (1,8%)	**14 (8.6%)**
**Age of first phrases (months)**							
Mean±SD	45.3±14.9	44.6±27.5	34.7±14.5	39.8±18.0	41.2±15.2	44.8±23.4	**39.8±18.0**
Min–Max	18–72	18–120	11–77	11–120	18–72	16–120	**11–120**
*Non delayed* (before 33 months old)	8 (4.9%)	9 (5.6%)	31 (19.1%)	48 (29.6%)	15 (9.5%)	4 (2.5%)	**48 (29.6%)**
*Delayed* (after 33 months old)	33 (20.4%)	11 (6.8%)	31 (19.1%)	75 (46.3%)	34 (21.0%)	14 (8.6%)	**75 (46.3%)**
*Not achieved*	12 (7.4%)	6 (3.7%)	21 (13.0%)	39 (24.1%)	15 (9.5%)	8 (4.9%)	**39 (24.1%)**

**Figure 1:**
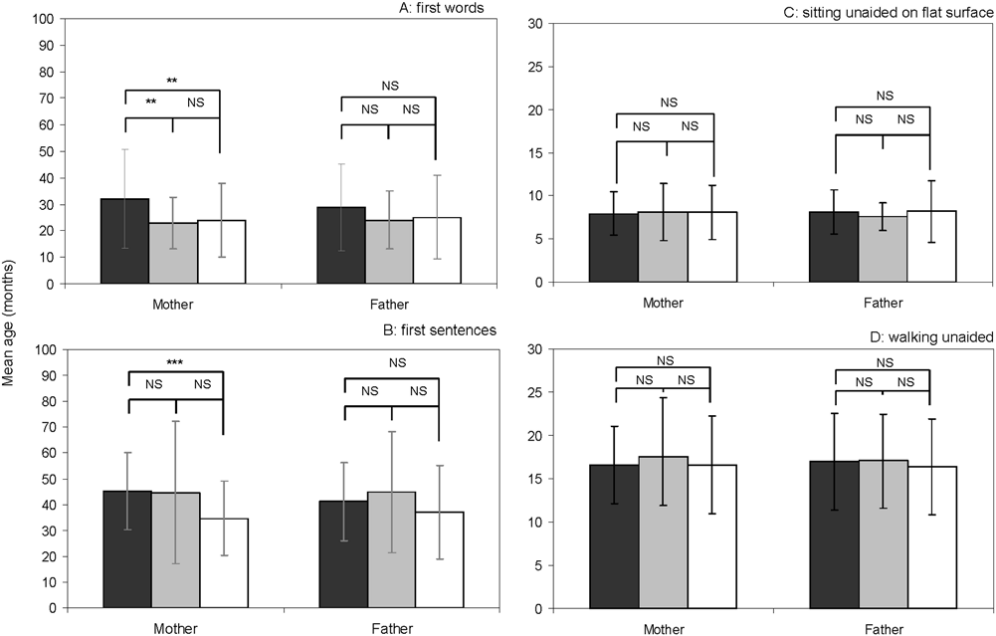
Mean age of the children for *first single words* (A), *first phrases* (B) *sitting unaided on flat surface* (C) and *walking unaided* (D) according to level of education of mothers and fathers. Error bars show standard deviation. Black bars represent the group of low level of education. Grey bars represent the group of mid level of education. White bars represent the group of high level of education. Level of significance: * p<0.05, ** p<0.01, *** p<0.001 (Mann Whitney U-test).

Fathers' levels of education did not influence significantly age of *first single words* (Kruskal-Wallis test: n = 147, H = 3.09, p = 0.21; Figure 1A) but mothers' levels of education did (Kruskal-Wallis test: n = 147, H = 7.12, p = 0.03; Figure 1A). Thus, *LES mothers'* children pronounced their *first single words* later than *HES mothers'* children and *MES mothers'* children (*X̅* = 32.1±18.5 months, *X̅* = 24.0±14.0 months, *X̅* = 23.0±9.8 months respectively, n_L_ = 48 n_H_ = 74 U = 3428 p = 0.01, n_L_ = 48 n_M_ = 25 U = 1942 p = 0.05; Figure 1A) *MES mothers'* children and *HES mothers'* children did not differ significantly (n_M_ = 25, n_H_ = 74, U = 1289, p = 0.76; Figure 1A).

**Figure 2:**
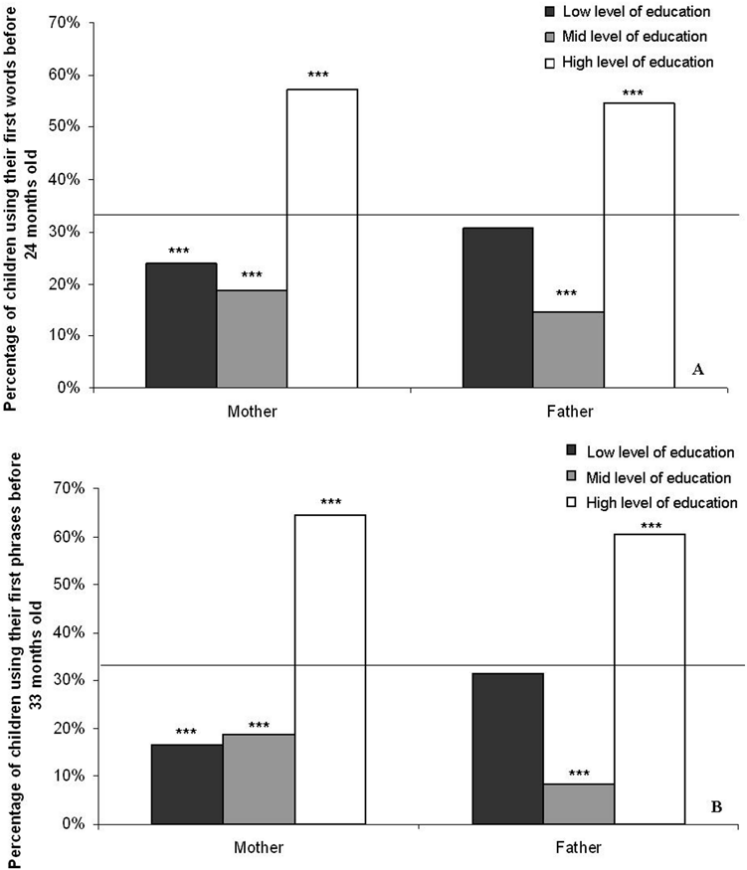
Mean percentages of children A: using their *first single words* before 24 months (*non delayed* group), B: using their *first phrases* before 33 months (*non delayed* group). Black bars represent the group of low level of education. Grey bars represent the group of mid level of education. White bars represent the group of high level of education. Black line indicates the mean percentage of children in each category according to level of education of mothers or fathers under random distribution. Values below the line indicate group the less represented in the category, values above the lines indicate group the more represented in the category. Level of significance: * p<0.05, ** p<0.01, *** p<0.001 (Chi square tests were made on real numbers).

Eighty-seven children (53.7%) of our cohort appeared to be *delayed*. The *non delayed* group and the *delayed* group differed according to levels of education of both mothers and fathers under random distribution (all χ^2^ tests p<0.001). Children of the *non delayed* group were mostly raised by *HES mothers* and *HES fathers*, whereas *LES fathers'* children, *MES mothers'* and *MES fathers'* children were less represented under random distribution (all χ^2^ tests p<0.001; Figure 2A). Children of the *delayed* group were mostly raised by *HES mothers'*, *LES mothers'*, and *LES fathers'* whereas *MES mothers'* and *MES fathers'* children were less represented under random distribution (all χ^2^ tests p<0.05).

#### Age of first phrases

One hundred and twenty-three children (75.9%) had uttered their *first phrases* and pronounced them on average at 39.8±18.0 months (min: 11; max: 120). Forty-eight children (29.6%) uttered their *first phrases* before 33 months (i.e. *non delayed*) and 75 children (46.3%) uttered their *first phrases* after 33 months (i.e. *delayed*). Thus, 39 children (24.1%) of the cohort had not pronounced their *first phrases* when they were assessed even though they were 78.2±48.2 months old (min: 37; max: 309) (Table 2).

Ages of *first phrases* did not differ significantly with fathers' level of education (Kruskal-Wallis test: n = 123, H = 4.01, p = 0.13; Figure 1B), but did differ significantly with mothers' level of education (Kruskal-Wallis test: n = 123, H = 12.38, p = 0.002; Figure 1B). Thus *LES mothers'* children uttered their *first phrases* significantly later than did *HES mothers'* children (*X̅* = 45.3±14.9 months, *X̅* = 34.7±14.5 months respectively, n_L_ = 41, n_H_ = 62, U = 2653, p<0.001; Figure 1B), whereas *MES mothers'* children were intermediate (n_M_ = 20, n_L_ = 41, U = 1355, p = 0.20 and n_M_ = 20, n_H_ = 62, U = 951, p = 0.19, respectively; Figure 1B).

One hundred and fourteen children (70.3%) of our cohort appeared to be *delayed*. The *non delayed* group and the *delayed* group differed significantly according to level of education of both mothers and fathers under random distribution (all χ^2^ tests p<0.001). Most children in the *non delayed* group were raised by *HES mothers'* and *HES fathers'*, while *LES fathers'* children, *MES mothers'* and *MES fathers'* children were less represented under random distribution (all χ^2^ tests p<0.001; Figure 2B). Most children in the *delayed* group were raised by *HES mothers'*, *LES mothers'*, and *LES fathers'* while *MES mothers'* and *MES fathers'* children were less represented under random distribution (all χ^2^ tests p<0.05).

#### Overall level of language

According to ADI-R, children could be divided into two categories of *overall level of language*. One hundred and four children (64.2%) appeared to have acquired sufficient verbal skills by the time they were assessed and they were then 109.0±56.9 months old (min: 37; max: 373), whereas 58 children (35.8%) used mostly single words or fewer than five words on a daily basis and were assessed when 77.0±42.5 months old (min: 37; max: 309).

A significant effect of the age was observed on the overall level of language (p = 0.01 for both mothers and fathers; Table 3) but both level of education of mothers and fathers and interaction with age did not have influence on the overall level of language (p>0.05; Table 3).

**Table 3:** Ordinal logistic regression of association between age of children at assessment and both mothers and fathers level of education (factors used both in independence and in interaction; with * in table).

	Global level of language			
	Mothers		Fathers	
	Odds ratio (95% CI)	p	Odds ratio (95% CI)	p
**Level of education**				
Low level	**Reference category**		**Reference category**	
Middle level	3.57 (0.21–61.75)	0.38	0.44 (0.02–9.77)	0.60
High level	2.12 (0.30–15.16)	0.45	3.75 (0.63–22.38)	0.15
**Age**	1.02 (1.00–1.04)	0.01	1.02 (1.01–1.04)	0.01
Level of education * Age				
Low level * Age	**Reference category**		**Reference category**	
Middle level * Age	0.99 (0.96–1.02)	0.44	1.01 (0.98–1.05)	0.48
High level * Age	0.99 (0.97–1.02)	0.57	0.99 (0.97–1.01)	0.22

Level of significance: p<0.05.

CI: confidence intervals.

#### Abnormality of development evident at or before 36 months

According to ADI-R, the *abnormality of development evident at or before 36 months* of children could be scored between 0 and 5. 149 children (91.9%), that is most children, were clearly impaired at assessment with scores of 3 and more.

The score of *abnormality of development evident at or before 36 months* differed according to both mothers (F(2,159) = 3.36, p = 0.037) and fathers level of education (F(2,159) = 3.96, p = 0.021). Thus *LES mothers* children had higher scores than *HES mothers* children (*X̅* = 4.189±0.942, *X̅* = 3.651±1.338 respectively, Tukey's test p<0.01) and than *MES mothers* children (*X̅* = 4.189±0.942, *X̅* = 3.885±1.071 respectively, Tukey's test p<0.01) whereas *HES mothers* children and *MES mothers* children did not differ. Thus *LES fathers* children had higher scores than *HES fathers* children (*X̅* = 4.231±1.142, *X̅* = 3.583±1.254 respectively, Tukey's test p<0.01) while *MES fathers* children did not differ (*X̅* = 4.031±1.083, *X̅* = 4.231±1.142, and *X̅* = 4.031±1.083, *X̅* = 3.583±1.254, both Tukey's test p>0.05).

### Sensori-motor development

Mother's and father's levels of education did not influence significantly the *age of sitting unaided on flat surface* (Kruskal-Wallis test: n = 159, H = 0.23, p = 0.89; H = 0.28, p = 0.86 respectively; Figure 1C), *the age of walking unaided* (Kruskal-Wallis test: n = 159, H = 0.69, p = 0.71; H = 0.81, p = 0.67 respectively; Figure 1D), *the age of bladder control acquisition during daytime* (Kruskal-Wallis test: n = 114, H = 1.49, p = 0.48; H = 0.35, p = 0.84 respectively), *the age of bladder control acquisition during the night* (Kruskal-Wallis test: n = 102, H = 2.90, p = 0.23; H = 0.35, p = 0.84 respectively) and *the age of bowel control acquisition* (Kruskal-Wallis test: n = 105, H = 2.66, p = 0.26; H = 1.18, p = 0.56 respectively). No significant difference was found between *LES*, *MES* and *HES mothers'* and *fathers'* children (all Mann Whitney tests p>0.05)

## Discussion

Our study of early characteristics of language development in a large sample of children with ASD revealed the influence of parents' level of education and a differential influence of mothers and fathers on these characteristics. In addition, general abnormalities appeared to be influenced by parents' level of education. Thus the language of children raised by high level of education parents developed earlier and first single words and first phrases were uttered earlier by children with high level of education mothers. Although some genetic transmission of cognitive abilities cannot be totally excluded at that stage [30], these results strongly suggest the importance of environmental factors, such as parental influence, on behavioural development of children with such disorders. However these influences clearly related to language as sensori-motor stages were not affected. This study constitutes, to our knowledge, the first demonstration of such an influence.

One could argue that this evaluation of dates of first words and phrases may be biased by the retrospective aspect of the survey: parents may not be sure of when these occurred. This is certainly true but was common for all classes of parents and therefore would not explain the differences observed. Also, the general features of language outputs in the ASD children studied here agree with previous reports showing that about half such a population never acquires functional language [31] and confirming that language impairments are one of the first signs of ASD [e.g. 23]. Our large sample shared global deficits with all the other populations studied, which may reveal shared biological sources. However, as in normal children population, inter-individual variation was high and, contrary to expectations from earlier studies [32], strongly associated with parents' socioeconomic status, included level of education. Thus, an earlier review [32] showed only 4 of 12 studies aiming to relate ASD and social class supported the possibility of such a link but concluded that social class was not a risk factor. Our findings suggest indeed that other risk factors are probably important as global deficits are found in children with ASD of parents from all levels of education (deficits are related to the overall level of language but not to class). However our results show that environmental factors such as parent's level of education may influence more refined aspects such as age of *first single words* or *first phrases*. Reports show that some behavioural traits in animals may be more open to environmental influences than others and that individual variations result from the interplay between genetic and environmental influences [2].

Because environmental factors may act on very precise aspects, only detailed studies such as our present study could reveal their influence. The current predominance of genetic models for psychiatric disorders may also explain that such aspects have been overlooked [11]. Our results emphasize the importance of remaining focused on this dual influence. As Robert [11] mentioned, “there is no such thing as a genome without a system”.

The fact that external factors, especially social environment, has been found to influence language characteristics is not surprising, language being “a social act” [33]. Social influences may help both humans and animals to overcome inhibitions, and to achieve exceptional learning in vocal communication processes [34]. Children need both communicative opportunities and a language model in order to develop language [35], [36]. For example, mother's and father's levels of education are significant predictors of child language [37], [38]. Recent studies suggest that children with ASD share an inherent basis with typical language learners in at least some aspects of language acquisition and that therefore delays might result more from social disinterest than from a core language disability [39]. Tager-Flusberg [40] suggested that language impairments may reflect the lack of attention of these children to their social environment. ASD children can be so unresponsive to voices and speech that they are first believed to be deaf [40].

Perceptual deficits may indeed exist as a consequence of impairments of voice processing in the STS central area [17], but social withdrawal and lack of social attention may well be involved in these central abnormalities [20]. Individual variations in language impairments may therefore reflect variations in social attention/involvement [41].

How could level of education, and more generally socioeconomic status, explain these differences? Socioeconomic status is a compound variable [35] that creates “different basic conditions of life at different levels of the social order” [42]. It involves education level of parents, their income, social network (other people encountered by children) and the individual effects of these components are not well known [43]. However socioeconomic status has a strong impact on typical language learners. High socioeconomic status mothers talk more to their children, use a more varied vocabulary, read books to their children more readily [44], [45]. According to Hoff [46] and Huttenlocker et al. [47], socioeconomic status-related differences in richness of maternal speech explain socioeconomic status differences in the development of young children's vocabulary and syntax (review in [35]). In our study, mothers' level of education appeared to have a major effect on the age of first words and phrases, showing that children with ASD, like normal children, might be sensitive to maternal inputs. Interestingly, fathers' level of education appeared also to have an effect, as being delayed or non delayed in the production of first words and phrases depended on both parents' level of education. Fathers' parenting behaviours have been shown to be predictive of young children's language development [48] and fathers' outputs have been shown to predict language scores of children [37]. Nevertheless, very few studies have investigated the influence of fathers' socioeconomics status on their language inputs to their children [37]. Our data showed that at least the level of education is probably important for children with ASD as well.

However, the processes involved in stimulating language outputs in children with ASD of high level of education parents remain unknown: could these processes include more perceptual stimulations, more triggering of social attention, enriched environments, more language inputs from family members and outside friends [41]? At this stage, the answer is unknown but, our study, which to our knowledge, demonstrates for the first time an impact of parents' level of education on language outputs of children with ASD, should trigger important new lines of thought and research. It suggests an openness of some traits to environmental conditions, and probably social influences that would reveal greater plasticity than expected in these children. The next crucial step involves understanding the processes at stake (social attention, perceptual experience, brain plasticity…?): is perception improved, selective attention developed, what aspects are crucial (book reading? focused language outputs?). The finding that more general “abnormalities of development” are also influenced suggests that environmental conditions, even though they cannot overcome the profound basic biologically-based impairments, may help improve a series of finer behavioural disturbances.

It is the first evidence that language development of children with ASD is at least in part under the influence of social factors. This study may trigger important new lines of thought and research (on the mechanisms underlying this influence; stimulate investigations on the exact links between parents' socioeconomic status and their language inputs to their children), help equilibrate social and purely biological perspectives regarding ASD, and brings new hopes for environmentally based therapies.
